# Systemic Interleukin-4 Administration after Spinal Cord Injury Modulates Inflammation and Promotes Neuroprotection

**DOI:** 10.3390/ph10040083

**Published:** 2017-10-24

**Authors:** Rui Lima, Susana Monteiro, José P. Lopes, Pedro Barradas, Natália L. Vasconcelos, Eduardo D. Gomes, Rita C. Assunção-Silva, Fábio G. Teixeira, Mónica Morais, Nuno Sousa, António J. Salgado, Nuno A. Silva

**Affiliations:** 1 Life and Health Sciences Research Institute (ICVS), School of Medicine, University of Minho, Campus de Gualtar, 4710-057 Braga, Portugal; id6527@alunos.uminho.pt (R.L.); susanamonteiro@med.uminho.pt (S.M.); jspdrlps@gmail.com (J.P.L.); a62398@alumni.uminho.pt (P.B.); n.l.vasconcelos80@gmail.com (N.L.V.); eduardogomes@med.uminho.pt (E.D.G.); ritasilva@med.uminho.pt (R.C.A.-S.); fabioteixeira@med.uminho.pt (F.G.T.); morais-monica@hotmail.com (M.M.); njcsousa@ecsaude.uminho.pt (N.S.); asalgado@med.uminho.pt (A.J.S.); 2 ICVS/3B’s—PT Government Associate Laboratory, Braga/Guimaraes, Portugal

**Keywords:** spinal cord injury, neuroprotection, immunomodulation, Interleukin-4, neuroimmunology

## Abstract

Traumatic spinal cord injury (SCI) causes dramatic disability and dysfunction in the motor, sensory and autonomic systems. The severe inflammatory reaction that occurs after SCI is strongly associated with further tissue damage. As such, immunomodulatory strategies have been developed, aimed at reducing inflammation, but also at shaping the immune response in order to protect, repair and promote regeneration of spared neural tissue. One of those promising strategies is the intraspinal administration of the cytokine interleukin-4 (IL-4) that was shown to promote a phenotype on specific immune cells associated with neuroprotection and repair. In this work, we evaluated if a systemic delivery of IL-4 for a 7-days period was also capable of promoting neuroprotection after SCI by analyzing different neural cells populations and motor recovery. IL-4 treatment promoted an elevation of the anti-inflammatory cytokine IL-10 in the serum both at 24 h and 7 days after injury. Locally, treatment with IL-4 led to a reduction on cells expressing markers associated with inflammation, CD11b/c and iNOS. Importantly, IL-4 treatment increased the neuronal markers βIII-tubulin and NeuN, and the oligodendrocyte marker O4, suggesting a neuroprotective effect. Moreover, 100% of the animals treated with IL-4 were able to recover weight support against only 33% of saline treated animals. Overall, these results show that systemic administration of IL-4 positively impacts different aspects of spinal cord injury, creating a more favorable environment for recovery to take place.

## 1. Introduction

Spinal cord injury (SCI) is a devastating neurological disorder that affects thousands of individuals each year. In the US alone it is estimated an annual incidence of 40 cases per million population and approximately 12,500 new SCI cases each year [1]. Despite the intense and interdisciplinary research being performed on SCI topic, an effective treatment for SCI is still lacking.

After the initial trauma a cascade of events, also known as “secondary events”, occur that contribute to further tissue damage and neurological deficits. As such, the acute phase after injury represents a particularly interesting time window for candidate therapies since spared neurons from the initial trauma can be protected from secondary events. Among these events is the excessive inflammatory response that comprises the activation of resident microglia and infiltrating leukocytes. These cells start to release several cytokines and reactive oxygen species allowing additional extravasation of leukocytes and further inflammation and tissue damage [2,3]. Thus, it is not surprising that for the last few decades many of the efforts were to design candidate therapies aiming at terminating with different aspects of inflammation [4,5]. In fact, the only pharmacological approach for use in clinical settings was the acute administration of methylprednisolone, a potent anti-inflammatory drug [6], although its efficacy is controversial and current guidelines recommendations support its discontinuation [7,8,9].

While it is clear that an excessive proinflammatory response is a detrimental component of SCI pathophysiology, it is also evident that immune cells are pivotal players for neural tissue repair and regeneration by coordinating local and systemic responses to injury [10,11,12,13]. Based on this premise, part of the research focus on SCI shifted from developing therapies to abrogate the inflammatory response to developing immunomodulatory strategies aiming at reducing excessive proinflammatory responses and promoting pro-regenerative immune phenotypes [14,15,16,17,18].

Interleukin-4 (IL-4) has emerged as an interesting therapy strategy in the context of SCI, due to its ability to promote M2-alternative activation of macrophages [18,19], a phenotype associated with neuroprotection and repair [20]. Indeed, impaired IL-4 signaling, such as in the case of IL-4 KO mice [12] or in aged microglia [21] was associated with poor spontaneous functional recovery after SCI. Conversely, the restoration of IL-4 signaling by adoptive transfer of CD4+ T cells was demonstrated to promote neuroprotection, axon regrowth and functional recovery after SCI in mice [12], highlighting the importance of this cytokine to SCI-recovery. In fact, a single intraspinal administration of IL-4 48 h after SCI was shown to be sufficient to skew macrophages and microglia into an M2 phenotype, and more importantly, was associated with improved functional recovery in SCI-mice [18]. This interesting finding prompted the question of whether a systemic delivery of IL-4, rather than intraspinal, could represent a neuroprotective therapy since this is a more convenient route of administration in clinical settings.

Therefore, in this work we aimed at exploring the long-term impact (8 weeks after injury) of an acute/sub-acute IL-4 treatment on different neural cell populations and on functional recovery in a rat model of contusion SCI. Specifically, we aimed to investigate if IL-4 delivered systemically (intraperitoneal rather than intraspinal) can be used as a neuroprotective therapy for SCI.

## 2. Results

### 2.1. IL-4 Treatment Increased Circulating Levels of the Anti-Inflammatory Cytokine IL-10 and Reduced Inflammatory Markers at the Spinal Cord of Injured Rats

**Figure 1:**
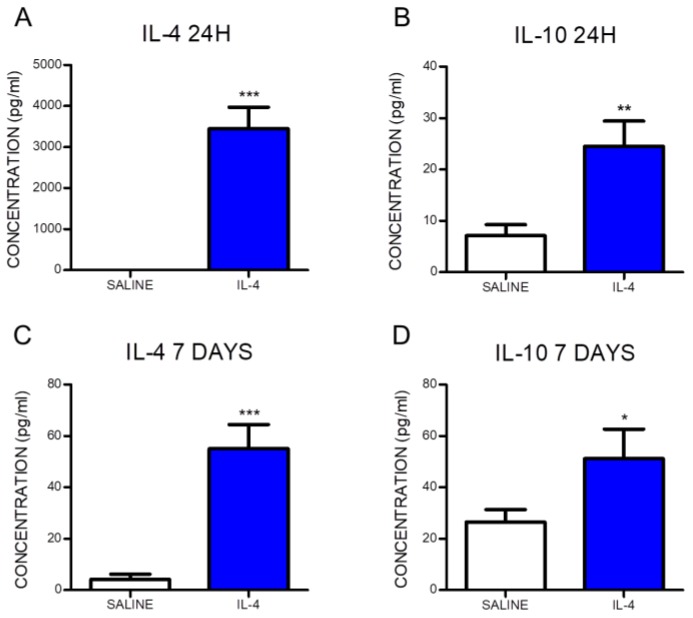
Interleukin-4 (IL-4) administered intraperitoneally reaches systemic circulation and promotes an elevation of the anti-inflammatory cytokine IL-10. The serum concentration of the cytokine IL-4 at 24 h (**A**) and 7 days (**C**) post-injury and IL-10 at 24 h (**B**) and 7 days (**D**) post-injury are significantly increased in the IL-4-treated rats. Values shown as mean ± SEM. * *p* < 0.05; ** *p* < 0.01; *** *p* < 0.001.

Blood samples were collected at 24 h and 7 days post-injury in order to confirm that the intraperitoneal administration of IL-4 was successfully reaching systemic bloodstream. Cytokine analysis was performed, revealing a significant increase in the levels of IL-4 in the serum from IL-4-treated animals when compared to the saline group (Figure 1A,C). Moreover, IL-4 treatment significantly promoted an elevation of the anti-inflammatory cytokine IL-10, both at 24 h and 7 days post-injury (Figure 1B,D). At 24 h post-injury, the IL-4 serum concentration in saline-treated animals was below detection limit while in the animals treated with IL-4 the concentration was 3451.2 ± 1165.5 pg/mL. At 7 days post-injury, the serum concentration of IL-4 was 13-fold higher in IL-4-treated rats than in saline-treated animals (IL-4: 55.0 ± 25.0 pg/mL; Saline: 4.2 ± 7.1 pg/mL). The anti-inflammatory cytokine IL-10 was detected at both time points for the two groups. However, 24 h post-injury the serum of IL-4-treated animals presented a 3.5-fold increase in IL-10 levels compared to saline-treated animals (IL-4: 24.5 ± 10.9 pg/mL; Saline: 7.1 ± 7.3 pg/mL). The higher concentration of IL-10 in IL-4-treated rats was also detected 7 days post-injury, with a 2-fold increase when comparing to saline-treated rats (IL-4: 51.1 ± 25.7 pg/mL; Saline: 26.5 ± 17.5 pg/mL).

Histological analysis was performed at the chronic phase (8 weeks post-injury) in order to assess the long-term impact of IL-4 treatment.

**Figure 2:**
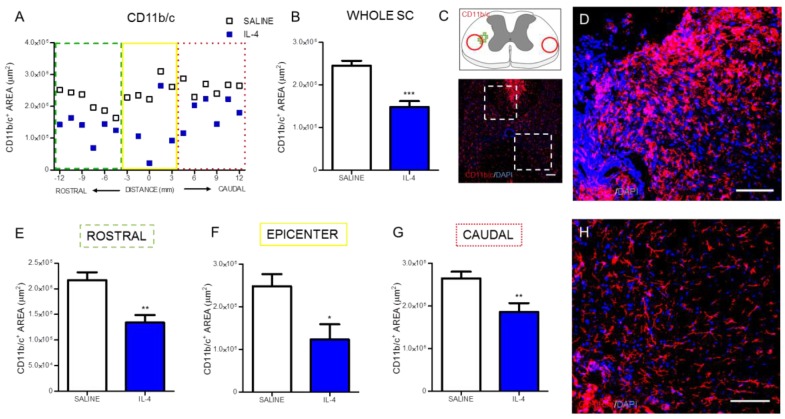
IL-4 treatment reduces the area of macrophages/microglia in the injured spinal cord. Distribution of the CD11b/c^+^ area along the rostrocaudal axis of the spinal cord (**A**). Quantification of CD11b/c^+^ area in the whole spinal cord revealed a significant reduction of macrophages in IL-4-treated rats (**B**); A significant reduction of CD11b/c^+^ area was also observed in the rostral (**E**); epicenter (**F**) and caudal (**G**) regions of the injured spinal cord. Schematic and low-magnification photomicrograph indicating areas where the analyses were performed (dashed lines) (**C**); Representative images of positive staining for macrophages/microglia of saline (**D**) and IL-4 treated (**H**) group. Values shown as mean ± SEM. * *p* < 0.05; ** *p* < 0.01; *** *p* < 0.001. Scale bar = 100 μm.

Macrophages/microglia were quantified through the expression of CD11b/c in two different regions of the spinal cord: the cavitation area and in spared tissue (Figure 2C).

Analysis of CD11b/c-positive cells revealed that IL-4 treatment significantly decreased the area occupied by macrophages (Figure 2A,B) on the spinal cord. The analysis of specific regions of the spinal cord demonstrated that the significant decrease of macrophages occurred both rostral (Figure 2E) and caudally to the epicenter (Figure 2G), as well as around the epicenter area (Figure 2F).

**Figure 3:**
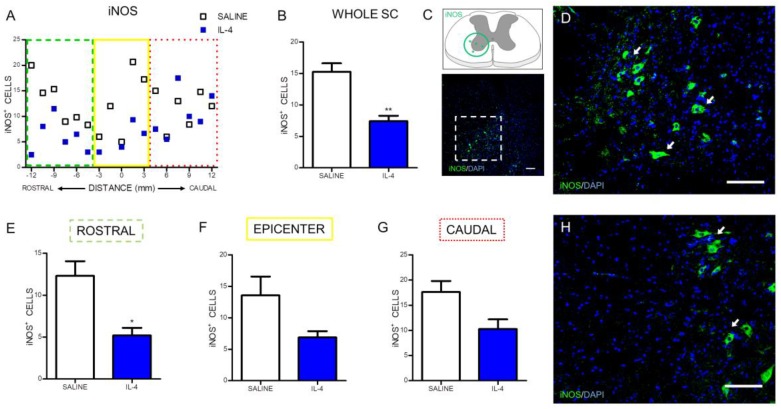
IL-4 treatment reduces the number of iNOS-expressing cells in the injured spinal cord. Distribution of iNOS^+^ cells along the rostrocaudal axis of the spinal cord (**A**); Quantification of iNOS^+^ cells in the whole spinal cord revealed a significant reduction of iNOS-producing cells in IL-4-treated rats (**B**); A significant reduction of iNOS-expressing cells was also observed in the rostral (**E**) area of the spinal cord but not at the epicenter (**F**) and in the caudal (**H**) regions. Schematic and low-magnification photomicrograph indicating areas where the analyses were performed (dashed lines) (**C**); Representative images of positive staining for iNOS^+^ cells of saline (**D**) and IL-4 treated (**H**) group. Values shown as mean ± SEM. * *p* < 0.05; ** *p* <0.01. Scale bar = 100 µm.

Besides the evaluation of the macrophages/microglia numbers, it was also analyzed the number of iNOS-positive cells, an enzyme associated with oxidative stress (Figure 3C). IL-4 treatment significantly reduced the iNOS-positive cells along the spinal cord when compared with saline treatment (Figure 3A,B). However, the analysis on specific regions of the spinal cord only revealed a statistically significant decrease of iNOS-positive cells rostrally to the epicenter (Figure 3E). Around (Figure 3F) and caudally (Figure 3G) to the epicenter it is possible to observe a clear trend for the decrease of iNOS-positive cells in the IL-4-treated group, however, no statistically significant differences were found.

**Figure 4:**
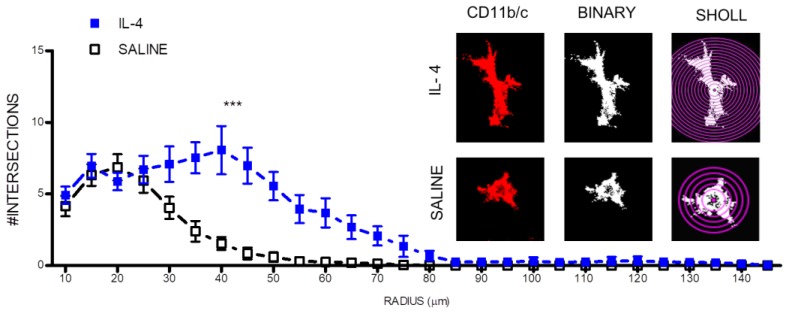
IL-4 treatment promotes a more ramified morphology (less active) on macrophages/microglia. On the right, microglia immunofluorescence images were transformed into binary and then analyzed with a sholl plot. Then, it was quantified the number of intersections with each sholl plot concentric circle. *** *p* < 0.001.

Interestingly, IL-4 treatment also led to morphological changes in macrophage/microglia cells. IL-4 treatment promoted a more ramified morphology in macrophage/microglia cells present at the lesion epicenter as shown by an increase on the number of intersecting ramification with concentric circles from a sholl plot (Figure 4).

### 2.2. IL-4 Treatment Increased the Number of Motor Neurons and Oligodendrocytes, while no Impact Was Observed on Astrocytes in the Spinal Cord of Injured Rats

We first assessed the neuroprotective effect of IL-4 on motor neurons. For that we quantified the number of motor neurons on the ventral horns using the neuronal marker NeuN and the area of expression of βIII-tubulin, a cytoskeletal protein and a major constituent of a neuron’s microtubules [22].

**Figure 5:**
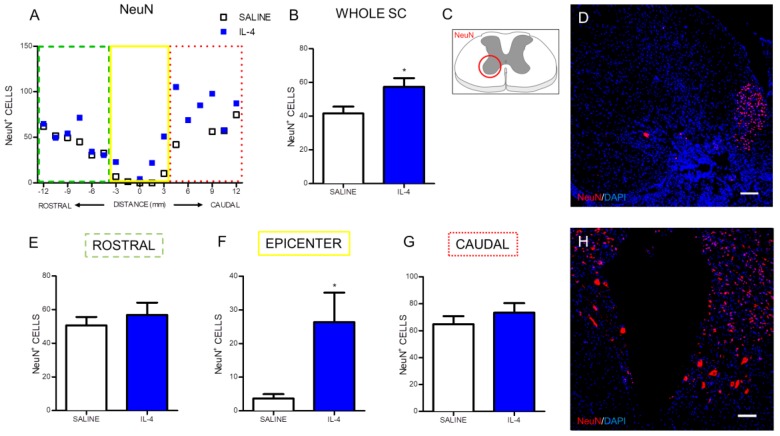
IL-4 treatment increases the number of motor neurons in the ventral horns. Distribution of the NeuN^+^ cells along the rostrocaudal axis of the spinal cord (**A**); Quantification of NeuN^+^ cells in the whole spinal cord revealed a significant increase of motor neurons in IL-4-treated rats (**B**); A significant increase of motor neurons was also observed at the epicenter region of the spinal cord (**F**); while in the rostral (**E**) and at the caudal (**G**) areas no differences could be observed. Schematic image indicating areas where the analyses were performed (**C**); Representative images of positive staining for motor neurons of saline (**D**) and IL-4 treated (**H**) group. Values shown as mean ± SEM. * *p* < 0.05. Scale bar = 100 µm.

Overall, IL-4 treatment led to an increase in the number of motor neurons in the ventral horns (Figure 5A,B). When the analysis was divided into rostral, epicenter and caudal region, it was observed that this increase of motor neurons was confined to the epicenter region (Figure 5E–G).

**Figure 6:**
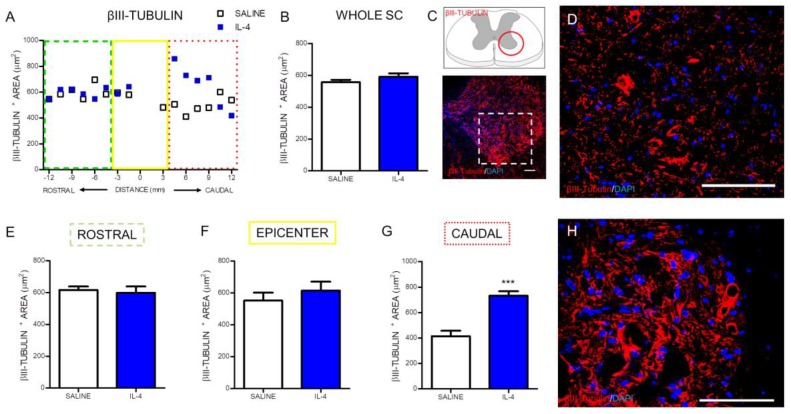
IL-4 treatment increases the area of staining for βIII-tubulin in the caudal part of the injured spinal cord. Distribution of βIII-tubulin positive area along the rostrocaudal axis of the spinal cord (**A**); Quantification of βIII-tubulin^+^ area in the whole spinal cord did not reveal any differences between saline or IL-4 treated rats (**B**) however a significant increase of βIII-tubulin area was observed in the caudal (**G**) area of the spinal cord of IL-4-treated rats; In the epicenter (**F**) and in the rostral (**E**) area no differences were observed. Schematic and low-magnification photomicrograph indicating areas where the analyses were performed (dashed lines) (**C**); Representative images of positive staining of neuronal cytoskeleton of saline (**D**) and IL-4 treated (**H**) group. Values shown as mean ± SEM. *** *p* < 0.001. Scale bar = 100 µm.

The quantification of the area occupied by βIII-tubulin-positive cells revealed a significant increase in the stained area in the caudal zone of the IL-4-treated rats’ spinal cords (Figure 6G). Although, the same effect was not verified when the analysis included the whole spinal cord (Figure 6B) or was performed on other specific regions of the spinal cord, such as the rostral (Figure 6E) or the epicenter (Figure 6F) zone.

**Figure 7:**
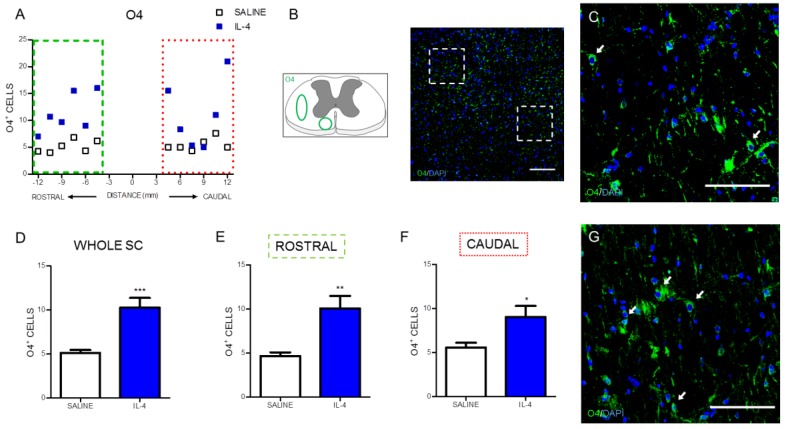
IL-4 treatment increases the number of oligodendrocytes in the injured spinal cord. Distribution of the O4^+^ cells along the rostrocaudal axis of the spinal cord (**A**); Quantification of O4^+^ cells in the whole spinal cord revealed a significant increase of oligodendrocytes in IL-4-treated rats (**D**); A significant increase of oligodendrocytes was also observed in the rostral (**E**) and caudal (**F**) regions of the injured spinal cord. Schematic and low-magnification photomicrograph indicating areas where the analyses were performed (dashed lines) (**B**); Representative images of positive staining for oligodendrocytes of saline (**C**) and IL-4 treated (**G**) group. Values shown as mean ± SEM. * *p* < 0.05; ** *p* < 0.01; *** *p* < 0.001. Scale bar = 100 µm.

The effect of IL-4 treatment on oligodendrocytes, the myelinating cells of the spinal cord neurons, was evaluated by quantifying O4, a marker for type I and II oligodendrocytes [23]. Overall, IL-4 treatment increased the number of oligodendrocytes (Figure 7A) on the injured spinal cord. Both the analysis to the whole spinal cord (Figure 7D) or to its specific rostral and caudal regions (Figure 7E,F) revealed a significant increase in the number of oligodendrocytes from IL-4-treated rats.

**Figure 8:**
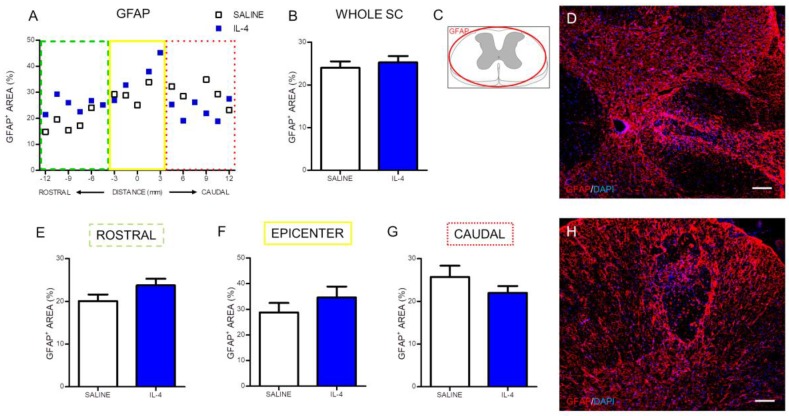
IL-4 treatment does not affect astrocyte numbers after SCI. Distribution of the percentage of GFAP^+^ area along the rostrocaudal axis of the spinal cord (**A**); The quantification of GFAP^+^ area either in the whole spinal cord (**B**) or in the specific regions (rostral (**E**); epicenter (**F**) and caudal (**G**)) did not reveal any differences in astrocytes’ numbers. Schematic image indicating areas where the analyses were performed (**C**); Representative images of positive staining for astrocytes of saline (**D**) and IL-4 treated (**H**) group. Values shown as mean ± SEM. Scale bar = 100 µm.

The effect of IL-4 on astrocytes was also evaluated using immunohistochemistry against the glial fibrillary acidic protein (GFAP). The positive stained area for GFAP was quantified along the rostral to caudal axis of the spinal cord (Figure 8A). The analysis demonstrated that IL-4 treatment did not alter GFAP-positive stained area in any region of the spinal cord (Figure 8).

### 2.3. IL-4 Treatment: The Effect on Motor Recovery and Lesion Size

**Figure 9:**
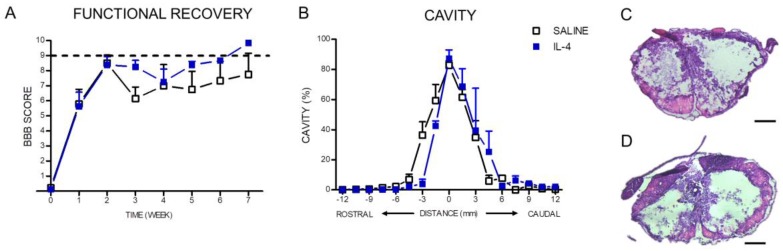
Despite the fact that no statistical differences were found between the Basso, Beattie and Bresnahan (BBB) score of each group, all IL-4 treated rats recovered weight support at week 7 post injury contrasting with only 33% of the saline group (**A**); Hematoxylin-staining revealed no differences on the lesion area extent (**B**); Representative images of hematoxylin–eosin staining of saline (**C**) and IL-4 treated (**D**) group. Values shown as mean ± SEM. Scale bar = 400 µm.

The Basso, Beattie and Bresnahan (BBB) score was applied in order to assess the locomotor behavioral recovery from SCI and was applied every week until week 7. Both saline and IL-4 treated group show a spontaneous improvement on the BBB score over time stabilizing after 2 weeks (Figure 9A). After 7 weeks (time of last BBB evaluation) the saline treatment-group had a BBB score of 8.5 ± 3.2 and the IL-4 treatment group a score of 9.6 ± 0.2 (Figure 9A). The mean between groups was not statistically significant, however, it is important to point out that 100% of the animals treated with IL-4 were able to recovery weight support against only 33% of saline treated animals.

The size of lesion tissue was quantified using hematoxylin–eosin staining. The analysis of the percentage of lesion tissue revealed that IL-4 treatment did not alter the percentage of lesion (Figure 9B).

## 3. Discussion

Here we showed that a scheme of repeated peritoneal injections of IL-4 for 7 days promotes neuroprotection after SCI. Specifically, an increase in motor neurons and oligodendrocytes was observed after 8 weeks post injury, without significant changes on astrocytes.

Interestingly, we describe the neuroprotective effects of IL-4 after SCI using a different animal model (rat versus mice) and a different route of administration (systemic versus intraspinal) from what was previously reported [18]. The observation that IL-4 neuroprotective effect is still achieved by systemic delivery and that this effect is conserved across at least these two rodent species highlights the therapeutic potential of this cytokine. The administration of specific cytokines to modulate the excessive inflammatory after SCI is a strategy tested by several groups. One example is the administration of IL-37, a cytokine from the IL-1 family that has recently been described as having anti-inflammatory effects, that has been shown to be neuroprotective after SCI by promoting enhanced functional recovery [24]. Another example of a cytokine tested after SCI is IL-25, a cytokine related with the development of type-2 responses. This cytokine has been shown to have detrimental effects after SCI [25]. Contrary to our approach, most of these candidate therapies using cytokines rely on local administration soon after the injury. Interestingly, the study using IL-25 has tested both local and repeated systemic administration and while local IL-25 administration had clear negative effects, its systemic repeated administration did not produce any detectable effect [25].

A central challenge on cytokine therapy is associated with the short half-life of cytokines in circulation. Since cytokines delivered in vivo may have a reduced half-life, we repeated peritoneal administration twice a day for 7-days and verified if this strategy was successful in raising IL-4 levels in circulation. We confirmed that not only IL-4 but also IL-10 was increased in the serum of treated rats.

The raised levels of this anti-inflammatory cytokine is particularly interesting since IL-10 has the ability to suppress cellular immunity and inhibit the synthesis and release of pro-inflammatory mediators [26,27] possibly counteracting the excessive inflammatory reaction seen after SCI. Indeed, the direct administration of IL-10 in animal models of SCI was demonstrated to promote neuroprotection, neuronal survival and functional improvements [28,29,30].

After confirming that IL-4 was in circulation during the acute/sub-acute phase after injury, we focused on the long-term (8 weeks after SCI) effects of IL-4 treatment on cellular and functional recovery. At first we assessed markers associated with inflammation and observed significant reduction of CD11b/c-positive cells (Figure 2) that may reflect reduced numbers of infiltrating macrophages and/or resident microglia and of iNOS-producing cells (Figure 3), an enzyme associated with oxidative activity and mainly induced by M1-macrophages. Reducing iNOS-producing cells may be protective for spinal cord injury as excessive iNOS expression in SCI is associated with oxidative damage and neuronal death [31,32,33,34,35]. Interestingly, macrophage/microglia changed morphology in response to IL-4 treatment from a more amoeboid state into a more ramified, a morphology associated with a less activated phenotype [36].

An important correlate of the reduced inflammatory response observed after IL-4 treatment is the increased numbers of motor neurons observed after treatment suggesting a neuroprotective effect of IL-4. In fact, additionally to the total number of motor neurons, we also observe an increase in βIII-tubulin stained area, possibly reflecting an enhanced structural preservation of motor neurons. However we cannot exclude that IL-4 treatment promoted axonal regeneration. Previous studies supported both an effect of IL-4 on protection and regeneration. For example, IL-4 was shown to promote motor neuron survival through STAT6 signaling in a model of peripheral nerve injury [37], while in dorsal root ganglion cells, IL-4 enhanced axonal regeneration through the stimulation of local neurotrophin secretion [38]. Walsh and colleagues demonstrated that the IL-4 receptor is expressed on spinal cord axons [12], so it is possible that the effects of IL-4 on axonal regeneration in peripheral nerve are also transversal to spinal cord axons.

Additionally we observed that beyond neuronal cells, IL-4 treatment increased the number of oligodendrocytes as shown by an increase in the numbers of O4-positive cells. Although also expressed in mature oligodendrocytes, O4 starts to be expressed in progenitors cells [39]. Therefore, a possible explanation for this increase in O4-positive cells induced by IL-4 treatment may be an increase in the formation of new oligodendrocytes. In line with this, is the study by Butovsky and co-workers showing that co-culturing neural precursors cells (NPCs) with microglia pre-exposed to IL-4 promoted oligodendrogenesis [40]. However, it is also known that activated macrophages/microglia may induce oligodendrocyte death and myelin damage through secreted factors [41], therefore, IL-4 treatment in our study may have protected existing mature oligodendrocytes, a fact supported by the IL-4-induced reduction in the numbers of macrophages/microglia observed in our study.

Our study demonstrated that IL-4 administration after a SCI, despite altering macrophages/microglia, oligodendrocytes and neurons, did not influence astrocytes. It is important to note that the role of astrocytes after a SCI is very complex and whether it has beneficial or detrimental effects is yet to be clearly demonstrated. Despite several studies showing that the astrocytic scar is a physical and chemical barrier against axonal regeneration [42,43] some other studies have shown a beneficial and crucial role of astrocytes after a SCI. For instance, the elimination of reactive astrocytes or, the prevention of their migration, and scar formation after injury resulted in a failure of blood-brain barrier repair accompanied by massive inflammatory cell infiltration and increased loss of neurons and oligodendrocytes leading to worse functional outcomes [44,45]. More recently, Anderson and colleagues [46] demonstrated that preventing, attenuating or ablating the astrocytic scar does not promote spontaneous axonal regeneration. On the contrary, they demonstrated that the astrocytic glial scar aids axonal regeneration [46].

Curiously, although IL-4 treatment had a protective effect in both neuronal and oligodendrocyte populations, this was not significantly reflected by the expected reduction of the size of the injured tissue. However, analysis of spared tissue does not take into account important factors, such as the presence of non-functional fibrotic tissue. Indeed, in the images of NeuN staining (Figure 5) it is possible to see that the saline group present intact tissue at the epicenter region, however, motor neurons were unable to survive, most likely due to neurodegeneration. Moreover, these differences in the functionality of the spared tissue are more likely correlated with the improvements observed in some features of locomotion. Indeed, we here demonstrated that similarly to what was previously demonstrated for a intraspinal IL-4 administration [18], a more clinically relevant administration of IL-4 (intraperitoneal injections) was also able to achieve an important landmark of motor recovery – the ability of rats to support their own weight. In fact, the BBB scores for the IL-4 group ranged between 9 and 10 meaning that while all the rats recovered weight support when stationary, some of the them were even able to perform occasional weight supported plantar stepping.

Although treatment with IL-4 led to significant neuroprotective effects, the recovery is still partial and far from complete perhaps because IL-4 only targets only some of the many aspects of this complex injury.

In this sense, IL-4 could be potentially used as part of a combinatory strategy either with other immunomodulatory therapies targeting, for example, adaptive immune cells [17] as well as with other regenerative strategies such as cell therapy [47], biomaterials [48] or other molecular therapies focused in neuroprotection or neuroregeneration.

## 4. Materials and Methods

### 4.1. Spinal Cord Injury Model and Treatment

Twelve Wistar Han female rats (14 weeks old, weighing 210–260 g) were used in this study. Animals were kept under standard laboratory conditions (12 h light: 12 h dark cycles, 22 °C, relative humidity of 55%, *ad libitum* access to standard food and water), and housed in pairs. Animal handling was carried out 3 days prior to surgery. Animals were subjected to a severe contusive SCI as previously described [49,50]. General anaesthesia was induced by an intraperitoneal injection (i.p.) of ketamine (100 mg/mL, Imalgene/Merial, Duluth, GA, USA) and medetomidine hydrochloride (1 mg/mL, Dormitor/Pfizer, New York, NY, USA) mixture, at a volume ratio of 1.5:1. Once anesthetized, animals received subcutaneous injections of the analgesic butorphanol (10 mg/mL, Butomidor/Richter Pharma AG, Wels, Austria), and the antibiotic enrofloxacin (5 mg/mL, Baytril/Bayer, Leverkusen, Germany). The fur was shaved from the surgical site and the skin disinfected with ethanol 70% and chlorohexidine. Surgical procedures were performed under sterile conditions. The animals were placed in a prone position and a dorsal midline incision was made at the level of thoracic spine (T5–T12). The paravertebral muscles were retracted and the spinous processes and laminar arc of T8 was removed, and the spinal cord exposed. The *dura* was left intact. A weight drop trauma model was used, that consisted in dropping a 10 g weight rod from a 20 cm height onto the exposed spinal cord [49]. The rod was guided through a stabilized tube that was positioned perpendicularly to the centre of the spinal cord. After the trauma, the muscles were sutured with Vicryl suture (Johnson and Johnson, New Brunswick, NJ, USA) and the incision closed with surgical staples (Fine Science Tools, Heidelberg, Germany). Anesthesia was reversed using atipamezole (5 mg/mL, Antisedan/Pfizer, New York, NY, USA). One-hour post-injury, animals were randomly divided into two experimental groups: IL-4 treatment (rat recombinant IL-4 (Kemprotec, Carnforth, UK) (0.35 µg/kg) and controls that received only vehicle (saline). The dosage selected was based on other studies using intraperitoneal injection of cytokines after a SCI [30,51]. Treatment and saline was administered i.p. every 12 h for 7 days to target the peak of macrophage infiltration [52].

Post-operative care for all rats included butorphanol (Richter Pharma AG, Wels, Austria) administration twice a day, for a five-day period as well as vitamins (Duphalyte, Pfizer, New York, NY, USA), saline, and enrofloxacin (Bayer, Leverkusen, Germany), twice a day for a 7-day period. Manual expression of bladders was performed twice a day until animals recovered spontaneous voiding. Body weight was monitored weekly as a parameter of general health of the animals. If a weight loss over 10% of body weight was detected, a high-calorie oral supplement (Nutri-Cal^®^) was administered daily.

### 4.2. Behavioral Assessment

The BBB locomotor rating scale [53] was used to evaluate functional recovery. Researchers performed all behavioral tests blindly to the treatment groups. The BBB test was performed three days post-injury and thereafter weekly for a 7-week period. A BBB score of 0 indicates no hindlimb movement. A BBB score of 1 through 8 indicates joint movement, but no weight support. A BBB score of 9 through 20 indicates an ability to support weight and use the limb for locomotion but with some degree of abnormality. A BBB score of 21 corresponds to the locomotion of a normal rat.

### 4.3. Serum Cytokine Analysis

After 24 h and 7-days post-injury, blood was collected from the tail and allowed to clot for 30 min before centrifugation (10 min at 10,000×*g*). Then, serum was collected and frozen at −80 °C. An enzyme-linked immunosorbent assay for IL-4 and IL-10 detection (Millipore) was used and the assay was performed as instructed by the supplier. Samples were analyzed in a MAGPIX Luminex’s xMAP^®^ instrument (Luminex, Austin, TX, USA).

### 4.4. Histological Assessment

Eight-weeks post-injury, animals were deeply anesthetized by an i.p. injection of sodium pentobarbital (200 mg/mL, Eutasil/Ceva Sante Animale, Libourne, France) and transcardially perfused with 100 mL of cold 0.9% saline followed by 300 ml of 4% paraformaldehyde (PFA) in 1× phosphate-buffered saline (PBS). A rough dissection of the vertebral column and spinal cord was performed and tissues were fixed in a solution of 4% PFA for 24 h (4 °C). The spinal cord was then dissected from the vertebral column and immersed in a cryoprotectant solution—30% sucrose, for 48 h at 4 °C. Afterwards, 2 cm length of spinal cord tissues, centered on the lesion, were submerged in optimal cutting temperature (OCT) embedding medium, frozen on dry ice, and stored at −20 °C. To minimize bias, each spinal cord was coded to keep the experimenter blind to the treatment. Cross-sections of 20 μm thickness were performed using a cryostat (Leica CM1900, LeicaBiosystems, Nussloch, Germany) and thaw-mounted onto charged microscope slides (Superfrost Plus, Thermo Scientific, Waltham, MA, USA). All histological procedures and evaluation were performed blindly to the treatment groups.

### 4.5. Hematoxylin–Eosin Staining

Tissue slides were stained for hematoxylin–eosin staining and then photographed with a stereology microscope (Zeiss Axioplan 2 Imaging, Jena, Germany) using a 2.5× objective. Evaluation of damaged tissue was performed on transverse sections (150 μm apart) along the rostrocaudal axis. The areas were manually traced and quantified using ImageJ software (National Institutes of Health, Bethesda, MD, USA).

### 4.6. Immunohistochemistry Protocol

For immunofluorescence staining, slices were washed with PBS, permeabilized with 0.2% Triton X-100 for 10 m and blocked with 5% fetal calf serum in 0.2% Triton X-100 for 30 m. Afterwards, the following primary antibodies were incubated overnight at room temperature (RT): mouse anti-CD11b/c for microglia and macrophages (1:100; Pharmingen, San Diego, CA, USA); rabbit inducible nitric oxide synthase (iNOS) (1:100; Millipore, Darmstadt, Germany), mouse anti-βIII-tubulin for neuronal cytoskeleton (1:1000; Promega, San Luis Obispo, CA, USA), mouse anti-O4 for oligodendrocytes (1:200, Millipore, Darmstadt, Germany), rabbit anti-GFAP for astrocytes (1:200; Dako Denmark, Glostrup, Danmark) and mouse anti-NeuN for neurons (1:200, Millipore, Darmstadt, German). The following day primary antibodies were then probed (2 h incubation) with the appropriate Alexa 594- or Alexa 488-conjugated secondary antibodies (1:1000; Invitrogen, Paisley UK). Sections were counterstained with DAPI for 30 m (1:1000; Sigma, Saint Louis, CA, USA) and mounted with Immu-Mount^®^ (Thermo Scientific, Waltham, MA, USA). Between steps, 5 washes with PBS (1×) were performed. For all immunofluorescence procedures, the appropriate negative controls were obtained by omission of the relevant primary antibody. Images were acquired using confocal point-scanning microscope, Olympus FV1000. All images were analyzed using ImageJ and FIJI software.

### 4.7. Immunofluorescence Analysis

Spinal cord immunostaining was analyzed by collecting photomicrographs every 150 μm both rostrally and caudally from the epicenter. The epicenter region was considered the area ranging from −300 μm and 300 μm surrounding the lesion epicenter. The most rostral area analyzed extended from −1200 μm to −300 μm from the lesion epicenter and the most caudal area analyzed extended from 300 μm to 1200 μm from the lesion epicenter. A section’s exclusion criteria for analysis were: shattered, cracked or folded sections or sections washed off during immunostaining procedure. After obtaining micrographs through confocal microscopy, the photos were opened with the Image J software. For cell counting, the multi-point tool was used. For positive area measurements, first the scale was determined and then the images were converted to 8 bits and were processed in the menu “make binary”. Finally, using the menu “analyze particles”, the software automatically calculated the areas occupied by each marker, using the dark background as contrast.

The immunofluorescence quantification in each photomicrograph was assessed by positive-cell counting (for the iNOS, NeuN and O4 markers) or positive staining area (for the CD11b/c, βIII-tubulin and GFAP area). Specifically, the analysis for the CD11b/c marker was performed in two random fields of each photomicrograph (Figure 2C). Since the staining for this marker in the epicenter region was heterogeneous due to the presence of a cavitation, the strategy was to select one field within the cavitation and other field outside the cavitation. Quantification of iNOS-positive cells was assessed in niches of positive cells in each section (Figure 3C). NeuN-positive cells were counted in the ventral horns of the gray matter (Figure 4C). βIII-tubulin positive area was measured on the ventral horns of the gray matter (Figure 5C). O4-positive cells were counted in two specific motor tracts, the vestibulospinal and the reticulospinal tracts Figure 6B) (the corticospinal tract was analyzed but no positive staining was found). Identification of tracts was according to the spinal cord atlas [54]. Data plotted in the graphs represent mean numbers (or area) per section. Data plotted in the graphs represent mean numbers (or area) per section. Finally, the GFAP-positive area was measured on the entire spinal cord slice using lower magnification (Figure 7C). Due to cavitation the GFAP-positive area is presented in percentage for the total spinal cord tissue.

### 4.8. Macrophages/Microglia Sholl Analysis

Z-stack confocal images of spinal cord section stained for CD11b/c were acquired using a confocal microscope and loaded into FIJI software. Images were thresholded to binary to include complete macrophage/microglia processes. A single macrophage/microglia cell was isolated by erasing surrounding processes from other cells, using the eraser tool. The longest process was drawn using the line segment tool. Using the sholl analysis function, the first shell was defined at 10 μm from the soma center (to exclude soma from the analysis) and each subsequent step at 5 μm. Analysis was performed using spinal cord sections representative of the lesion epicenter. Two to three macrophage/microglia cells per section were randomly selected for analysis. Exclusion criteria for macrophage/microglia cell selection for analysis were: incomplete processes or macrophage/microglia with processes near to the margins. Number of intersections with each concentric shell was automatically calculated by the software considering a linear profile.

### 4.9. Statistical Analysis

Statistical analysis was performed using GraphPad Prism 6.00 software. The normality of the data was evaluated by the Kolmogorov-Smirnov normality tests. When the equal variances criterion was not met, Welch correction was applied.

Data from macrophage/microglia sholl analysis and BBB test was assessed by a two-way ANOVA test. Differences between groups were compared with the post hoc Bonferroni test.

Immunofluorescence and cytokine concentration data were analyzed using the Student’s *t*-test or Mann-Whitney according normality results. Statistical significance was defined for *p* < 0.05 (95% confidence level). Data are shown as mean +/− standard error (SEM).

## 5. Conclusions

Our results show that IL-4 treatment for 7-days led to a reduced inflammatory profile and enhanced neuronal and oligodendrocytes populations while having no significant impact in astrocytes. Moreover, IL-4 treatment improved some features of the motor behaviour of SCI animals. The successful impact of IL-4 treatment in histological and functional aspects of SCI highlights the potential of this therapy on integrating other therapeutic approaches. However, more pre-clinical studies are needed to determine if IL-4 can enter in clinical trials in the future to be used in established clinical protocols directed towards SCI.

## References

[1] National Spinal Cord Injury Statistical Center (2005). Facts and figures at a glance. J. Spinal Cord Med..

[2] Popovich P.G., Wei P., Stokes B.T. (1997). Cellular inflammatory response after spinal cord injury in sprague-dawley and lewis rats. J. Comp. Neurol..

[3] Hausmann O.N. (2003). Post-traumatic inflammation following spinal cord injury. Spinal Cord.

[4] Arnold S.A., Hagg T. (2011). Anti-inflammatory treatments during the chronic phase of spinal cord injury improve locomotor function in adult mice. J. Neurotrauma.

[5] Mabon P.J., Weaver L.C., Dekaban G.A. (2000). Inhibition of monocyte/macrophage migration to a spinal cord injury site by an antibody to the integrin αD: A potential new anti-inflammatory treatment. Exp. Neurol..

[6] Bracken M.B., Shepard M.J., Collins W.F., Holford T.R., Young W., Baskin D.S., Eisenberg H.M., Flamm E., Leo-Summers L., Maroon J. (1990). A randomized, controlled trial of methylprednisolone or naloxone in the treatment of acute spinal-cord injury. Results of the second national acute spinal cord injury study. N. Engl. J. Med..

[7] Evaniew N., Noonan V.K., Fallah N., Kwon B.K., Rivers C.S., Ahn H., Bailey C.S., Christie S.D., Fourney D.R., Hurlbert R.J. (2015). Methylprednisolone for the treatment of patients with acute spinal cord injuries: A propensity score-matched cohort study from a canadian multi-center spinal cord injury registry. J. Neurotrauma.

[8] Resnick D.K. (2013). Updated guidelines for the management of acute cervical spine and spinal cord injury. Neurosurgery.

[9] Silva N.A., Sousa N., Reis R.L., Salgado A.J. (2014). From basics to clinical: A comprehensive review on spinal cord injury. Prog. Neurobiol..

[10] Shechter R., London A., Varol C., Raposo C., Cusimano M., Yovel G., Rolls A., Mack M., Pluchino S., Martino G. (2009). Infiltrating blood-derived macrophages are vital cells playing an anti-inflammatory role in recovery from spinal cord injury in mice. PLoS Med..

[11] Gadani S.P., Walsh J.T., Lukens J.R., Kipnis J. (2015). Dealing with danger in the cns: The response of the immune system to injury. Neuron.

[12] Walsh J.T., Hendrix S., Boato F., Smirnov I., Zheng J., Lukens J.R., Gadani S., Hechler D., Gölz G., Rosenberger K. (2015). MHCII-independent cCD4+ T cells protect injured cns neurons via IL-4. J. Clin. Investig..

[13] Donnelly D.J., Popovich P.G. (2008). Inflammation and its role in neuroprotection, axonal regeneration and functional recovery after spinal cord injury. Exp. Neurol..

[14] Popovich P.G., Longbrake E.E. (2008). Can the immune system be harnessed to repair the CNS?. Nat. Rev. Neurosci..

[15] Chiu C.W., Huang W.H., Lin S.J., Tsai M.J., Ma H., Hsieh S.L., Cheng H. (2016). The immunomodulator decoy receptor 3 improves locomotor functional recovery after spinal cord injury. J. Neuroinflammation.

[16] Ma S.F., Chen Y.J., Zhang J.X., Shen L., Wang R., Zhou J.S., Hu J.G., Lu H.Z. (2015). Adoptive transfer of M2 macrophages promotes locomotor recovery in adult rats after spinal cord injury. Brain. Behav. Immun..

[17] Hu J.G., Shi L.L., Chen Y.J., Xie X.M., Zhang N., Zhu A.Y., Jiang Z.S., Feng Y.F., Zhang C., Xi J. (2016). Differential effects of myelin basic protein-activated Th1 and Th2 cells on the local immune microenvironment of injured spinal cord. Exp. Neurol..

[18] Francos-Quijorna I., Amo-Aparicio J., Martinez-Muriana A., Lopez-Vales R. (2016). IL-4 drives microglia and macrophages toward a phenotype conducive for tissue repair and functional recovery after spinal cord injury. Glia.

[19] Ghosh M., Xu Y., Pearse D.D. (2016). Cyclic amp is a key regulator of m1 to M2a phenotypic conversion of microglia in the presence of Th2 cytokines. J. Neuroinflammation.

[20] Kigerl K.A., Gensel J.C., Ankeny D.P., Alexander J.K., Donnelly D.J., Popovich P.G. (2009). Identification of two distinct macrophage subsets with divergent effects causing either neurotoxicity or regeneration in the injured mouse spinal cord. J. Neurosci..

[21] Fenn A.M., Hall J.C., Gensel J.C., Popovich P.G., Godbout J.P. (2014). IL-4 signaling drives a unique arginase+/IL-1β+ microglia phenotype and recruits macrophages to the inflammatory CNS: Consequences of age-related deficits in IL-4Rα after traumatic spinal cord injury. J. Neurosci..

[22] Alexander J.E., Hunt D.F., Lee M.K., Shabanowitz J., Michel H., Berlin S.C., Macdonald T.L., Sundberg R.J., Rebhun L.I., Frankfurter A. (1991). Characterization of posttranslational modifications in neuron-specific class III β-tubulin by mass spectrometry. Proc. Natl. Acad. Sci. USA.

[23] Sommer I., Schachner M. (1981). Monoclonal antibodies (O1 to O4) to oligodendrocyte cell surfaces: An immunocytological study in the central nervous system. Dev. Biol..

[24] Coll-Miro M., Francos-Quijorna I., Santos-Nogueira E., Torres-Espin A., Bufler P., Dinarello C.A., Lopez-Vales R. (2016). Beneficial effects of IL-37 after spinal cord injury in mice. Proc. Natl. Acad. Sci. USA.

[25] Dooley D., Lemmens E., Ponsaerts P., Hendrix S. (2016). Interleukin-25 is detrimental for recovery after spinal cord injury in mice. J. Neuroinflammation.

[26] Moore K.W., de Waal Malefyt R., Coffman R.L., O’Garra A. (2001). Interleukin-10 and the interleukin-10 receptor. Annu. Rev. Immunol..

[27] Howard M., O’Garra A., Ishida H., de Waal Malefyt R., De Vries J. (1992). Biological properties of interleukin 10. J. Clin. Immunol..

[28] Bethea J.R., Nagashima H., Acosta M.C., Briceno C., Gomez F., MARCILLO A.E., Loor K., Green J., Dietrich W.D. (1999). Systemically administered interleukin-10 reduces tumor necrosis factor-alpha production and significantly improves functional recovery following traumatic spinal cord injury in rats. J. Neurotrauma.

[29] Zhou Z., Peng X., Insolera R., Fink D.J., Mata M. (2009). IL-10 promotes neuronal survival following spinal cord injury. Exp. Neurol..

[30] Brewer K.L., Bethea J.R., Yezierski R.P. (1999). Neuroprotective effects of interleukin-10 following excitotoxic spinal cord injury. Exp. Neurol..

[31] Kaushal V., Koeberle P.D., Wang Y., Schlichter L.C. (2007). The Ca^2+^-activated K^+^ channel KCNN4/KCa3. 1 contributes to microglia activation and nitric oxide-dependent neurodegeneration. J. Neurosci..

[32] Kaushal V., Schlichter L.C. (2008). Mechanisms of microglia-mediated neurotoxicity in a new model of the stroke penumbra. J. Neurosci..

[33] Chatzipanteli K., Garcia R., Marcillo A.E., Loor K.E., Kraydieh S., Dietrich W.D. (2002). Temporal and segmental distribution of constitutive and inducible nitric oxide synthases after traumatic spinal cord injury: Effect of aminoguanidine treatment. J. Neurotrauma.

[34] López-Vales R., García-Alías G., Forés J., Navarro X., Verdú E. (2004). Increased expression of cyclo-oxygenase 2 and vascular endothelial growth factor in lesioned spinal cord by transplanted olfactory ensheathing cells. J. Neurotrauma.

[35] Pearse D., Chatzipanteli K., Marcillo A., Bunge M., Dietrich W. (2003). Comparison of inos inhibition by antisense and pharmacological inhibitors after spinal cord injury. J. Neuropathol. Exp. Neurol..

[36] Rostam H., Reynolds P.M., Alexander M.R., Gadegaard N., Ghaemmaghami A.M. (2017). Image based machine learning for identification of macrophage subsets. Sci. Rep..

[37] DeBoy C.A., Xin J., Byram S.C., Serpe C.J., Sanders V.M., Jones K.J. (2006). Immune-mediated neuroprotection of axotomized mouse facial motoneurons is dependent on the IL-4/STAT6 signaling pathway in cd4+ t cells. Exp. Neurol..

[38] Gölz G., Uhlmann L., Lüdecke D., Markgraf N., Nitsch R., Hendrix S. (2006). The cytokine/neurotrophin axis in peripheral axon outgrowth. Eur. J. Neurosci..

[39] Warrington A., Pfeiffer S. (1992). Proliferation and differentiation of O4+ oligodendrocytes in postnatal rat cerebellum: Analysis in unfixed tissue slices using anti-glycolipid antibodies. J. Neurosci. Res..

[40] Butovsky O., Ziv Y., Schwartz A., Landa G., Talpalar A.E., Pluchino S., Martino G., Schwartz M. (2006). Microglia activated by IL-4 or IFN-γ differentially induce neurogenesis and oligodendrogenesis from adult stem/progenitor cells. Mol. Cell. Neurosc..

[41] Probert L., Eugster H.-P., Akassoglou K., Bauer J., Frei K., Lassmann H., Fontana A. (2000). TNFR1 signalling is critical for the development of demyelination and the limitation of T-cell responses during immune-mediated cns disease. Brain.

[42] Silver J., Schwab M.E., Popovich P.G. (2015). Central nervous system regenerative failure: Role of oligodendrocytes, astrocytes, and microglia. Cold Spring Harb. Perspect. Biol..

[43] Busch S.A., Silver J. (2007). The role of extracellular matrix in CNS regeneration. Curr. Opin. Neurobiol..

[44] Faulkner J.R., Herrmann J.E., Woo M.J., Tansey K.E., Doan N.B., Sofroniew M.V. (2004). Reactive astrocytes protect tissue and preserve function after spinal cord injury. J. Neurosci..

[45] Okada S., Nakamura M., Katoh H., Miyao T., Shimazaki T., Ishii K., Yamane J., Yoshimura A., Iwamoto Y., Toyama Y. (2006). Conditional ablation of STAT3 or SOCS3 discloses a dual role for reactive astrocytes after spinal cord injury. Nat. Med..

[46] Anderson M.A., Burda J.E., Ren Y., Ao Y., O’Shea T.M., Kawaguchi R., Coppola G., Khakh B.S., Deming T.J., Sofroniew M.V. (2016). Astrocyte scar formation aids central nervous system axon regeneration. Nature.

[47] Silva N.A., Gimble J.M., Sousa N., Reis R.L., Salgado A.J. (2013). Combining adult stem cells and olfactory ensheathing cells: The secretome effect. Stem Cells Dev..

[48] Assunção-Silva R.C., Gomes E.D., Sousa N., Silva N.A., Salgado A.J. (2015). Hydrogels and cell based therapies in spinal cord injury regeneration. Stem Cells Int..

[49] Noble L.J., Wrathall J.R. (1985). Spinal cord contusion in the rat: Morphometric analyses of alterations in the spinal cord. Exp. Neurol..

[50] Vasconcelos N.L., Gomes E.D., Oliveira E.P., Silva C.J., Lima R., Sousa N., Salgado A.J., Silva N.A. (2016). Combining neuroprotective agents: Effect of riluzole and magnesium in a rat model of thoracic spinal cord injury. Spine J..

[51] Plunkett J.A., Yu C.-G., Easton J.M., Bethea J.R., Yezierski R.P. (2001). Effects of interleukin-10 (IL-10) on pain behavior and gene expression following excitotoxic spinal cord injury in the rat. Exp. Neurol..

[52] Beck K.D., Nguyen H.X., Galvan M.D., Salazar D.L., Woodruff T.M., Anderson A.J. (2010). Quantitative analysis of cellular inflammation after traumatic spinal cord injury: Evidence for a multiphasic inflammatory response in the acute to chronic environment. Brain.

[53] Basso D.M., Beattie M.S., Bresnahan J.C. (1995). A sensitive and reliable locomotor rating scale for open field testing in rats. J. Neurotrauma.

[54] Anderson C.R., Ashwell K.W.S., Collewijn H., Conta A., Harvey A., Heise C., Hodgetts S., Holstege G., Kayalioglu G., Keast J.R. (2009). The spinal cord: A christopher and dana reeve foundation text and atlas. The Spinal Cord.

